# The OPTIMIST study: optimisation of cost effectiveness through individualised FSH stimulation dosages for IVF treatment. A randomised controlled trial

**DOI:** 10.1186/1472-6874-12-29

**Published:** 2012-09-18

**Authors:** Theodora C van Tilborg, Marinus JC Eijkemans, Joop SE Laven, Carolien AM Koks, Jan Peter de Bruin, Gabrielle J Scheffer, Ron JT van Golde, Kathrin Fleischer, Annemieke Hoek, Annemiek W Nap, Walter KH Kuchenbecker, Petra A Manger, Egbert A Brinkhuis, Arne M van Heusden, Alexander V Sluijmer, Arie Verhoeff, Marcel HA van Hooff, Jaap Friederich, Jesper MJ Smeenk, Janet Kwee, Harold R Verhoeve, Cornelis B Lambalk, Frans M Helmerhorst, Fulco van der Veen, Ben Willem J Mol, Helen L Torrance, Frank JM Broekmans

**Affiliations:** 1 Department of Reproductive Medicine and Gynaecology, University Medical Centre Utrecht, University of Utrecht, Utrecht, The Netherlands; 2 Julius Centre for Health Sciences and Primary care, University Medical Centre Utrecht, Utrecht, The Netherlands; 3 Department of Obstetrics and Gynaecology, Division of Reproductive Medicine, Erasmus Medical Centre, Rotterdam, The Netherlands; 4 Department of Obstetrics and Gynaecology, Maxima Medical Centre, Veldhoven, The Netherlands; 5 Department of Obstetrics and Gynaecology, Jeroen Bosch Hospital, Den Bosch, The Netherlands; 6 Department of Obstetrics and Gynaecology, Gelre Hospital, Apeldoorn, The Netherlands; 7 Department of Obstetrics and Gynaecology, Maastricht University Medical Centre, University of Maastricht, Maastricht, The Netherlands; 8 Department of Obstetrics and Gynaecology, University Medical Centre St Radboud, Nijmegen, The Netherlands; 9 Department of Obstetrics and Gynaecology, University Medical Centre Groningen, University of Groningen, Groningen, The Netherlands; 10 Department of Obstetrics and Gynaecology, Rijnstate Hospital, Arnhem, The Netherlands; 11 Department of Obstetrics and Gynaecology, Isala Clinics, Zwolle, The Netherlands; 12 Department of Obstetrics and Gynaecology, Diakonessen Hospital Utrecht, Utrecht, The Netherlands; 13 Department of Obstetrics and Gynaecology, Meander Medical Centre, Amersfoort, The Netherlands; 14 Department of Obstetrics and Gynaecology, St Antonius Hospital, Nieuwegein, The Netherlands; 15 Department of Obstetrics and Gynaecology, Wilhelmina Hospital, Assen, The Netherlands; 16 Department of Obstetrics and Gynaecology, Maasstad Hospital, Rotterdam, The Netherlands; 17 Department of Obstetrics and Gynaecology, St Fransiscus Hospital, Rotterdam, The Netherlands; 18 Department of Obstetrics and Gynaecology, Gemini Hospital, Den Helder, The Netherlands; 19 Department of Obstetrics and Gynaecology, St Elisabeth Hospital, Tilburg, The Netherlands; 20 Department of Obstetrics and Gynaecology, St Lucas Andreas Hospital, Amsterdam, The Netherlands; 21 Department of Obstetrics and Gynaecology, Onze Lieve Vrouwe Hospital, Amsterdam, The Netherlands; 22 Department of Obstetrics and Gynaecology, VU University Medical Centre, Amsterdam, The Netherlands; 23 Department of Obstetrics and Gynaecology, Leids University Medical Centre, University of Leiden, Leiden, The Netherlands; 24 Department of Obstetrics and Gynaecology, Academic Medical Centre, University of Amsterdam, Amsterdam, The Netherlands

**Keywords:** Ovarian reserve, Antral follicle count, IVF, Individualised FSH stimulation dosages, Live birth rate

## Abstract

**Background:**

Costs of in vitro fertilisation (IVF) are high, which is partly due to the use of follicle stimulating hormone (FSH). FSH is usually administered in a standard dose. However, due to differences in ovarian reserve between women, ovarian response also differs with potential negative consequences on pregnancy rates. A Markov decision-analytic model showed that FSH dose individualisation according to ovarian reserve is likely to be cost-effective in women who are eligible for IVF. However, this has never been confirmed in a large randomised controlled trial (RCT). The aim of the present study is to assess whether an individualised FSH dose regime based on an ovarian reserve test (ORT) is more cost-effective than a standard dose regime.

**Methods/Design:**

Multicentre RCT in subfertile women indicated for a first IVF or intracytoplasmic sperm injection cycle, who are aged < 44 years, have a regular menstrual cycle and no major abnormalities at transvaginal sonography. Women with polycystic ovary syndrome, endocrine or metabolic abnormalities and women undergoing IVF with oocyte donation, will not be included. Ovarian reserve will be assessed by measuring the antral follicle count. Women with a predicted poor response or hyperresponse will be randomised for a standard versus an individualised FSH regime (150 IU/day, 225-450 IU/day and 100 IU/day, respectively). Participants will undergo a maximum of three stimulation cycles during maximally 18 months. The primary study outcome is the cumulative ongoing pregnancy rate resulting in live birth achieved within 18 months after randomisation. Secondary outcomes are parameters for ovarian response, multiple pregnancies, number of cycles needed per live birth, total IU of FSH per stimulation cycle, and costs. All data will be analysed according to the intention-to-treat principle. Cost-effectiveness analysis will be performed to assess whether the health and associated economic benefits of individualised treatment of subfertile women outweigh the additional costs of an ORT.

**Discussion:**

The results of this study will be integrated into a decision model that compares cost-effectiveness of the three dose-adjustment strategies to a standard dose strategy. The study outcomes will provide scientific foundation for national and international guidelines.

**Trial registration:**

NTR2657

## Background

In vitro fertilisation (IVF) is the treatment of last resort for many subfertile couples and a very costly one, partly due to the use of expensive drugs, i.e. gonadotrophins, needed for controlled ovarian hyperstimulation (COH) [1]. COH is an essential part of IVF which is needed to obtain a reasonable yield of oocytes which can then be fertilised in vitro. In clinical practice physicians often rely on their clinical experience and judgement when selecting an appropriate starting dose of follicle stimulation hormone (FSH). There is neither international nor nationwide consensus about COH programmes. Defining the optimal dose of FSH to retrieve an acceptable number of oocytes remains complicated. The problem herewith is that women show marked differences in their ovarian reserve and, as a consequence, in their ovarian response to medication [2,3]. Women who respond poorly to ovarian stimulation have poorer pregnancy prospects than women with a normal ovarian response [4]. Frequently, the occurrence of an insufficient response will urge the clinician to cancel the cycle which is obviously very stressful to the couple and may lead to withdrawal from further treatment. In a subsequent stimulation cycle higher dosages of FSH are usually given, although these may also be inadequate, leading to another cancellation of the cycle or even to hyperresponse with risk of ovarian hyperstimulation syndrome (OHSS). The result is that the poor responder may go through a series of treatment cycles with an overall poor prospect for pregnancy.

On the other end of the spectrum, women with a hyperresponse to ovarian stimulation are at risk of increased patient discomfort, cycle cancellation and development of OHSS. This condition may lead to severe illness requiring hospitalisation and intensive care with thromboembolic phenomena or multiple organ failure which are potentially life threatening complications. Mild and moderate forms of OHSS occur in respectively 20–33% and 3–6% of all ovarian stimulation cycles, while the severe form of the syndrome has been reported to occur as frequently as 0.1–2% [5].

In view of the tight relationship between hyperresponse and OHSS, prevention of hyperresponse is one of the key elements in preventing OHSS. Accurate prediction of ovarian response is now possible by using an ovarian reserve test (ORT) [6]. Various ORTs are available, of which basal FSH is the oldest whereas the antral follicle count (AFC) and serum level of anti-Müllerian hormone (AMH) have been introduced more recently. Previous systematic reviews by our group have shown that both AFC and AMH provide an optimal balance between sensitivity and specificity, are superior to basal FSH, and are able to predict ovarian response to COH, allowing for studies on the value of adjustment of the FSH dosage based on these parameters [6-10]. It should be emphasised that our reviews showed that ORTs can predict ovarian response after COH, but not the occurrence of pregnancy after IVF treatment.

Previous research has shown that dose adjustment after ORTs resulted in a higher rate of normal ovarian response and significantly higher pregnancy rates [11]. However, this study had several weaknesses. First of all, the power calculation was not based on the outcome ‘pregnancy’ but on the outcome ‘appropriate response’. Furthermore, participants with possible extremes in ovarian response were excluded, FSH dosage was only fixed until stimulation day 8, and the used algorithm was complicated and impractical for daily clinical use. Before definitive conclusions can be drawn about dose adjustment after ORTs, these results need to be confirmed in a large randomised controlled trial (RCT). Based upon these data, we hypothesize that the use of an ORT as predictor of ovarian response followed by an individualised FSH regime, will lead to a reduction in the occurrence of an inappropriate ovarian response, a reduction in the amount of cycle cancellations, reduction in the number of withdrawals from treatment, reduction in the occurrence of OHSS, reduction in the application of cycles with poor prospects for success, improvement in overall pregnancy rates and improvement of overall cost-effectiveness of IVF programs.

## Methods

### Study design

The OPTIMIST trial is a nationwide multicentre RCT. The cohort will consist of 1,500 women screened for ovarian reserve by an AFC prior to IVF or intracytoplasmic sperm injection (ICSI) treatment. Two RCTs are embedded in this cohort. Based on the AFC, women will be classified into four categories; AFC less than 8 (predicted poor responders), AFC 8-10 (predicted low-normal responders), AFC 11-15 (predicted normal responders) or AFC above 15 (predicted hyperresponders). RCT 1 will contain 300 women with an AFC below 11; RCT 2 will contain 300 women with an AFC of more than 15. A total of 25 academic and non-academic centres in the Netherlands will participate. An economic analysis is incorporated to assess the cost-effectiveness of an ORT and subsequent individualised FSH dosage in IVF or ICSI treatment. Inclusion was started in May 2011. This study will be conducted in accordance with the principles of the Declaration of Helsinki and in accordance with Good Clinical Practice. The study protocol has been approved by the Institutional Review Board (IRB) of the University Medical Center Utrecht (MEC 10-273) and by the board of directors of all participating centres.

### Sample size calculation

Based on the expected gain in pregnancy rate resulting in live birth as reported in the study by Popovic-Todorovic et al. [12], we expect an increase in pregnancy rate over three IVF or ICSI cycles of 15% (from 25% to 40%) in the predicted poor responders (RCT 1). To demonstrate that such a difference will occur, we need to include 300 couples in RCT 1 (alpha-error of 0.05 and a power of 80%, two-sided test). If we expect a positive effect of dose adjustment in both the predicted poor responders (RCT 1) and predicted hyperresponders (RCT 2), the total sample size of 600 women, in both RCTs, allows the detection of an increase in pregnancy rate from 30% after conventional treatment to 41% in the dose adjustment group (alpha-error of 0.05 and a power of 80%, two-sided test). If the net effect of dose adjustment on pregnancy rate is less than 11%, it is unlikely that an ORT and dose adjustment is cost-effective. The assumed 30% success rate in the conventional group in the latter calculation is higher than the assumed 25% success rate in the conventional group of solely RCT 1, as RCT 1 only contains women with an expected poor response. RCT 2 adds women with a predicted hyperresponse, who are expected to have higher pregnancy rates. For RCT 2 an increase in pregnancy rate from 35% to 42% over three cycles is expected. With 300 participants the power to detect this difference is 24%.

In our Individual Patient Data meta-analysis on 2,300 patients in 13 studies, we found that 25% had and AFC below 6, 18% had an AFC between 6 and 9, 33% had an AFC between 10 and 15 and 24% had an AFC of more than 15 [12].If we assume that 20% of the couples will have a low or low-normal ovarian reserve, and are therefore eligible for the predicted poor responder trial, we need to include a cohort of 1,500 women for ovarian reserve screening in order to achieve the necessary number of participants for the two trials. This will then allow a sample size of just over 300 women for RCT 1.

A sample size calculation for the cost effectiveness analysis is not provided. It is generally accepted that this type of sample size is not provided because the uncertainty of the cost economy ratio is largely determined by the variation in the efficacy (the denominator of the ratio) and can therefore not be performed at a realistic level.

### Recruitment, consent and randomisation

**Figure 1:**
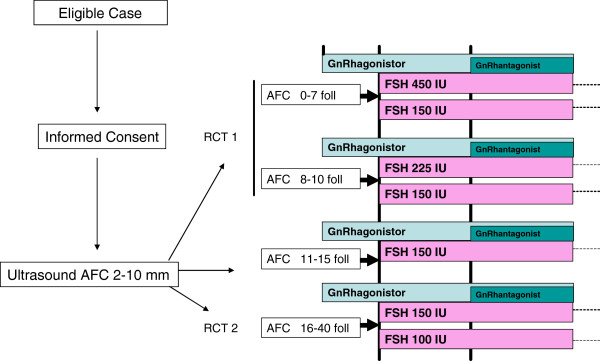
Study procedure.

Eligible women with an indication for a first IVF or ICSI treatment cycle or a first IVF or ICSI treatment cycle after birth of a child will receive oral and written information from their attending physician. The investigator will explain the study fully and in sufficient detail to allow the women to make an informed decision whether to participate or not. If a woman is willing to participate, written informed consent will be obtained. Based on AFC classification women will be entered into one of the four AFC classes (see Figure 1). Participants, excluding the predicted normal responders, will be randomly allocated to a standard or individualised FSH regime. All predicted normal responders will receive the standard dosage. Randomisation will be performed centrally by a web-based randomisation program and will be stratified by centre.

### Study population

All subfertile women indicated for a first IVF or ICSI cycle, who are aged < 44 years, have a regular menstrual cycle and no major uterine or ovarian abnormalities at TVS are considered to be eligible for participation. Women with polycystic ovary syndrome (PCOS) and (uncorrected) endocrine or metabolic abnormalities, as well as women undergoing IVF after oocyte donation, will not be included.

### Study procedures

The AFC will be measured to assess ovarian reserve capacity. The AFC will be performed in the early follicular phase (cycle day 1–3) of the stimulation cycle by using a standard TVS based measurement and count of all ovarian follicles sized 2–10 mm in both ovaries, performed by experienced physicians trained in applying this ultrasound test. On the same day serum will be obtained for post hoc analysis of endocrine ovarian reserve tests. Based on the result of the AFC women will either receive a standard or elevated FSH dosage (RCT 1) or a standard or reduced FSH dosage (RCT 2). All predicted normal responders will receive the standard dose regime. Recombinant or urinary FSH will be used for ovarian stimulation. Stimulation with the assigned FSH dose will be initiated on cycle day 2 or 3. Dose adjustments in the course of the stimulation cycle are not allowed. A gonadotrophin-releasing hormone (GnRH) agonist (long or flare-up protocol) or antagonist co-treatment protocol will be used for luteinizing hormone (LH) peak suppression. The participating hospitals will apply only one of the methods of LH suppression for all participants included in the study (GnRH analogue stratification). Final oocyte maturation will be achieved by administration of human chorionic gonadotrophin (hCG) when three or more follicles of 17 mm are present. Oocyte retrieval will be carried out 34–36 hours after hCG administration. Embryo transfer will be performed 3–5 days after ovum pick- up. Luteal phase supplementation will consist of 600 mg micronized progesterone in three separate dosages starting one day after oocyte retrieval and continued until 18 days after ovum pick-up. All participants will undergo a maximum of three IVF or ICSI cycles during a treatment period of maximally 18 months.

#### Experimental group

All women in the experimental group will receive an individualised FSH dose based on their AFC. If women in RCT 1 are randomised for dose adjustment they will use 450 IU FSH/day if the AFC is below 8 and 225 IU FSH/day if the AFC is 8-10. Women in RCT 2 will receive 100 IU FSH/day if they are randomised for dose adjustment. Participants will be subjected to the assigned dosage in the subsequent cycles. In RCT 1, dose adjustments are not allowed in a subsequent cycle. For women in RCT 2, dose adjustment in the second or third cycle is allowed, and can consist of a step of 25 IU FSH/day, if there is a poor response or hyperresponse (poor response is defined as the retrieval of less than five oocytes or the cancellation of a stimulation cycle because less than two follicles above 12 mm in diameter are observed on ultrasound; hyperresponse is defined as the retrieval of more than 15 oocytes or cancellation of a stimulation cycle because more than 20 follicles over 12 mm in diameter are growing and estradiol levels exceed 11.700 pmol/L (= 3187,08 ng/L) or if more than 30 follicles over 12 mm are growing).

#### Control group

All women in the control group will receive a standard FSH dose of 150 IU/day, independent of the AFC classification. They will be subjected to the assigned dosage in the subsequent cycles. However, based on the response in preceding cycles, a dose adjustment in the second and third cycle is allowed, but cannot exceed a step of 50 IU FSH/day, if there has been a poor response or hyperresponse (as defined above).

### Withdrawal of individual patients

Subjects can abandon the study at any time for any reason if they wish to do so without any consequences. The investigator can decide to withdraw a subject from the study for urgent medical reasons.

#### Outcome measures

The primary study outcome is the cumulative pregnancy rate achieved within 18 months after randomisation resulting in live birth. Pregnancies can be obtained in treatment cycles with fresh embryos as well as in subsequent cryo/thaw cycles. Spontaneous pregnancies between treatment cycles will also be taken into account.

The data will be integrated in a cost-effectiveness analysis. Differentiation will be made between direct medical costs (all health care sector costs), direct non-medical costs (costs outside the health care sector that are affected by health status or health care) and indirect costs of the fertility treatment (costs of sick leave due to fertility treatment). For medical costs, the process of care will be divided into two cost stages (cost of fertility treatment, cost of pregnancy), which can reoccur if women have repeat treatment cycles within the study period. These costs will be computed from the period of inclusion to the end of follow-up (18 months).

Direct non-medical and indirect costs may be generated if women are absent from paid work, either for visiting the fertility clinic during IVF or ICSI treatment or due to sick leave associated with physical or psychological side-effects of this treatment. The Health and Labour Questionnaire [13] will be used to document absence from paid work.

Fertility health care utilisation consists of hospital visits, TVS, gonadotrophins, endocrine laboratory tests, ovum pick-up, IVF or ICSI laboratory work and hospital care. Volumes of health care resource use will be measured alongside the clinical study in several participating centres by using a checklist.

Secondary study outcomes consist of the number of retrieved oocytes, the occurrence of poor response or hyperresponse, OHSS grade 2/3, the rate of cycle cancellation, the number of multiple pregnancies and total IU of FSH used per stimulation cycle.

#### Statistical analysis

SPSS and Excel will be used to perform the statistical analysis. A probability (p) of less than 0.05 will be considered to be significant. Data will be presented for all arms of the study group. Data will be expressed as means ± standard deviation and proportions or rates. The analysis will be by intention to treat.

Descriptive analysis will be used to describe the primary and secondary outcome variables and to compare these outcome variables among the treatment arms of the two RCTs. Comparisons between the two arms of the randomised group will be done by applying Chi/square testing to crude rates of cumulative implantation and ongoing pregnancy and by using life table analysis to account for the factor of time to implantation or pregnancy. The relative contribution of predictive factors of success in any of the treatment arms, such as female age and duration of subfertility will be assessed by univariate and multivariate logistic regression and Cox regression.

### Economic analysis

From a societal perspective, we will perform an economic analysis alongside the cohort study and clinical trials. The data on cancellation rates, FSH consumption and pregnancy rate from the cohort study (900 couples) and the two RCTs (300 patients per study) will be integrated in a cost-effectiveness analysis. We will build on previous work that we have done for CVZ (College Voor Zorgverzekeringen; College of Health Insurances). In the analysis, we will assess the cost-effectiveness of using an ORT for dose adjustments in poor responders ór hyperresponders only and using an ORT for dose adjustment in both poor responders and hyperresponders, as compared to current practice where no ORT is used and standard FSH doses are given.

The study design will enable us to compare the costs and effects of the following strategies:

I. IVF or ICSI without an ORT for three cycles with a standard start dose (reference strategy)

II. ORT followed by IVF or ICSI with dose adjustment in women with predicted hyperresponse and predicted poor response

III. ORT followed by IVF or ICSI and dose reduction in women with predicted hyperresponse only

IV. ORT followed by IVF or ICSI and dose adjustment in women with predicted poor response only

The key question in the economic evaluation is to assess whether the health and associated economic benefits of individualised treatment of subfertile women in terms of increased ongoing pregnancy rates and reduced rates of hyperstimulation, outweigh the additional costs of an ORT. The economic evaluation will be designed as a cost-effectiveness analysis with the costs per pregnancy resulting in live birth within 18 months as the primary outcome measure. Cost-effectiveness of each strategy will therefore be expressed as costs per live birth. The incremental cost-effectiveness ratio (ICER) of each individualised dose-adjustment strategy as compared to a strategy with standard FSH dose will be estimated as the ratio between difference in costs between strategies and the difference in pregnancy rates, and reflects the extra costs required to obtain one additional live birth. If in the predicted poor response group individualised FSH treatment following an ORT increases the live birth rate without affecting OHSS rates, individualised treatment is the dominant strategy. If in the predicted hyperresponse group individualised FSH treatment following an ORT reduces the rate of OHSS, without affecting live birth rates, individualised treatment is the dominant strategy.

## Discussion

The number of patients starting IVF or ICSI treatment in the Netherlands annually is approximately 7,000 and they undergo 16,000 IVF or ICSI cycles a year [14]. Women with a poor response or hyperresponse comprise 30- 55.8% of the population [11,15]. Bouwman et al. [1] showed that hormonal stimulation is the most expensive part of IVF and ICSI treatment.

In modern society, many women delay childbearing, despite intensive and costly efforts to increase awareness of the unavoidable ovarian failure associated with advanced age. In the Netherlands, the age at which a woman delivers her first child has increased from 25.5 years (1980) to 29.4 years (2011) making Dutch women amongst the oldest mothers in the world [16]. As a result of this delayed child bearing, the most frequent indication for assisted reproductive techniques such as IVF and ICSI is now unexplained subfertility in couples with increased female age, in which diminished ovarian reserve is usually the cause. As a consequence, the potential value of an ORT is increasing.

A recently published Markov decision-analytic model showed that individualisation of the FSH dose according to ovarian reserve is likely to be cost-effective in women who are eligible for IVF or ICSI treatment [17]. The use of an ORT followed by individualised FSH regimens can have a large impact on current IVF practice. The advantages may include improvement of quality of life for the patient, reduction of harm associated with conventional IVF, increased effectiveness and reduction of costs.

The results of this study will be integrated into a decision model that compares cost-effectiveness of the three dose-adjustment strategies to the standard FSH dose strategy. The costs and the live birth rates of these strategies will be assessed. The study outcomes will provide scientific foundation for national and international guidelines.

## Abbreviations

AFC: Antral follicle count; AMH: Anti-Müllerian hormone; COH: Controlled ovarian hyperstimulation; FSH: Follicle stimulating hormone; GnRH: Gonadotrophin- releasing hormone; hCG: Human chorionic gonadotrophin; ICER: Incremental cost-effectiveness ratio; ICSI: Intracytoplasmic sperm injection; IVF: In vitro fertilisation; LH: Luteinizing hormone; OHSS: Ovarian hyperStimulation syndrome; ORT: Ovarian reserve test; PCOS: Polycystic ovary syndrome; RCT: Randomised controlled trial; TVS: Trans vaginal sonography.

## Competing interests

The authors declare that they have no competing interests.

## Authors’ contributions

TT is responsible for the overall logistical aspects of the trial and drafted the manuscript. HT and FB have contributed to the development of the protocol and study design. FB applied for a grant. FB and HT have overall responsibility for the trial. ME is responsible for the sample size calculation and will be involved in the statistical analysis. JL, CK, JB, GS, RG, KF, AH, AN, WK, AM, EB, AvH, AS, AV, MH, JF, JS, JK, HV, CL, FH, FV and BM are responsible for implementation of the study and inclusion of eligible patients. All authors read and approved the final manuscript.

## Pre-publication history

The pre-publication history for this paper can be accessed here:

http://www.biomedcentral.com/1472-6874/12/29/prepub
